# Idiopathic Orbital Pseudotumor: A Rare Case Report

**DOI:** 10.7759/cureus.41602

**Published:** 2023-07-09

**Authors:** Ammar W Baltoyour

**Affiliations:** 1 Ophthalmology, Dhahran Eye Specialist Hospital, Dhahran, SAU

**Keywords:** case report, magnetic resonance imaging, corticosteroid treatment, blurry vision, orbital pseudotumor

## Abstract

Orbital pseudotumor is a rare inflammatory condition affecting the orbit of the eye. It has diverse clinical manifestations. Although its exact etiology remains unknown, it is believed to involve an immune-mediated response. A 42-year-old male presented to the ophthalmology clinic with progressively worsening symptoms in his left eye, including pain, swelling, and blurry vision. He had no history of trauma or recent illness. Initial discomfort had escalated over three weeks. The patient had controlled hypertension but no known allergies. Examination showed eyelid edema, erythema, and mild anterior chamber cell, and flare in the left eye. Magnetic resonance imaging revealed orbital soft tissue enhancement, extraocular muscle thickening, and optic nerve involvement. Laboratory results showed elevated inflammatory markers. A diagnosis of orbital pseudotumor was made. The patient was treated with oral corticosteroids, resulting in symptom improvement and regression of inflammatory changes on follow-up. Orbital pseudotumor is a complex condition with diverse clinical manifestations. Its diagnosis requires a comprehensive approach involving clinical evaluation, imaging studies, and laboratory investigations.

## Introduction

Orbital pseudotumor, also known as an idiopathic orbital inflammatory syndrome, is a rare inflammatory condition affecting the orbit of the eye. The inflammation associated with orbital pseudotumor can involve various anatomical locations and structures, including the extraocular muscles, glands, and connective tissues. Orbital pseudotumor has diverse clinical manifestations, including eye pain, swelling, visual disturbances, and proptosis [1]. Although its exact etiology remains unknown, it is believed to involve an immune-mediated response. The diagnosis of orbital pseudotumor is challenging, as it requires the exclusion of other orbital pathologies through a combination of clinical evaluation, imaging studies, and laboratory investigations [1]. Treatment typically involves systemic corticosteroids, but alternative immunosuppressive agents and biological therapies have also shown promise [2]. This case report aims to present a detailed clinical case of orbital pseudotumor, highlighting the diagnostic and therapeutic challenges encountered and discussing the management approach.

## Case presentation

A 42-year-old male presented to the ophthalmology clinic with a three-week history of progressively worsening symptoms in his left eye, including pain, swelling, and blurry vision. He denied any history of trauma or recent illness. The symptoms initially started as mild discomfort but gradually intensified over time.

The patient reported experiencing left eye pain, swelling, and blurry vision for the past three weeks, with a progressive worsening of symptoms. He denied experiencing fever, chills, or weight loss. There were no associated upper respiratory tract infection symptoms, joint pain, or morning stiffness. No other focal neurological deficits or symptoms were reported.

The patient had a history of well-controlled hypertension for the past five years, managed with antihypertensive medication. He had no known drug or environmental allergies. Visual acuity in the right eye was 20/20, while in the left eye, it was 20/40. External examination of the left eyelid revealed significant edema and erythema. The conjunctiva showed no signs of chemosis or injection. The cornea appeared clear without epithelial defects or infiltrates. Pupillary examination demonstrated equal and reactive pupils, with no relative afferent pupillary defect. Extraocular motility was full in all directions of gaze. Intraocular pressure measurements were within the normal range, with the right eye at 15 mmHg and the left eye at 17 mmHg. No remarkable findings were observed in the anterior segment of the right eye. However, mild anterior chamber cells and flare were noted in the left eye. Fundus examination revealed normal findings of the optic disc, macula, and peripheral retina in both eyes.

**Figure 1:**
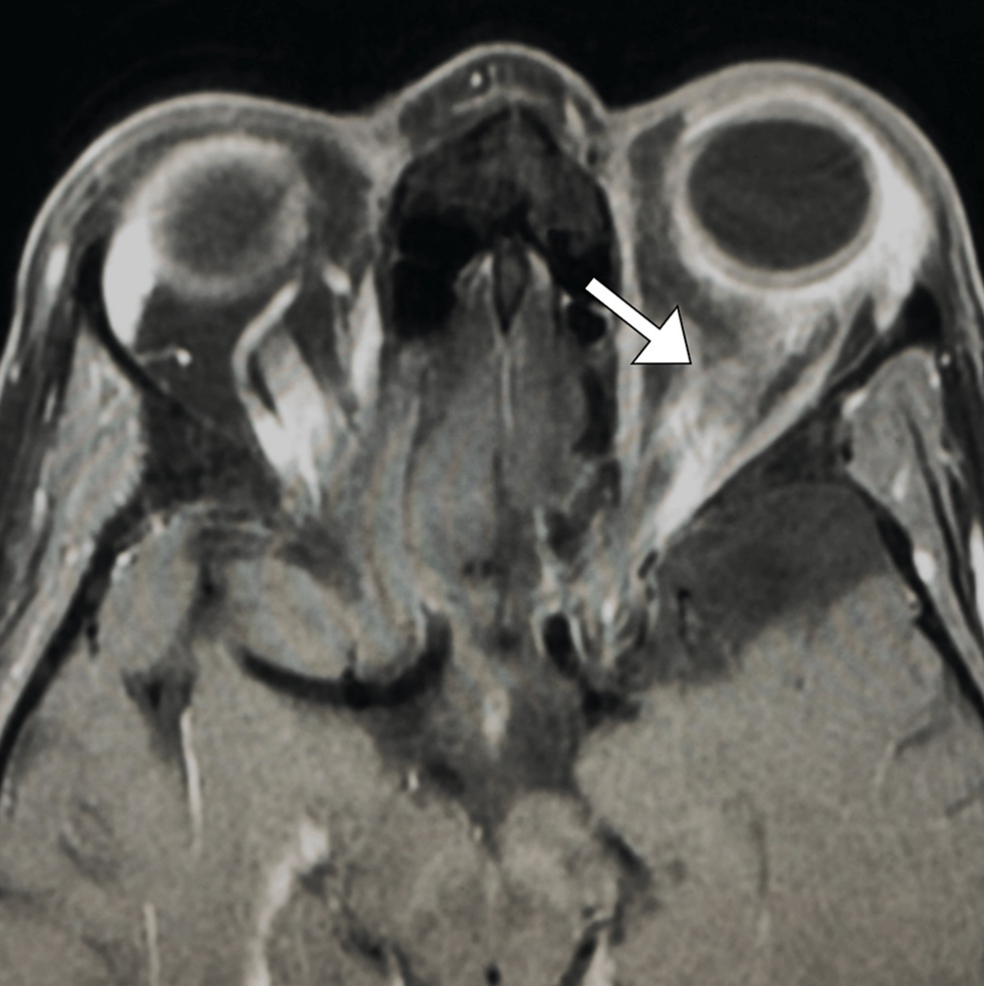
Axial MRI image with contrast revealing left retrobulbar soft tissue abnormalities, optic nerve enhancement, and mild swelling (arrow). MRI: magnetic resonance imaging

Magnetic resonance imaging (MRI) of the orbits with contrast was performed, revealing diffuse enhancement of orbital soft tissues with thickening of extraocular muscles and infiltration of orbital fat. There was also mild swelling and enhancement of the optic nerve (Figure 1).

Laboratory investigations showed a complete blood count within normal limits. The erythrocyte sedimentation rate (ESR) was elevated at 40 mm/hr (normal range: 0-20 mm/hr), and the C-reactive protein (CRP) was elevated at 12 mg/L (normal range: 0-8 mg/L). The thyroid-stimulating hormone level was within normal limits.

Based on the clinical presentation, imaging findings, and laboratory results, the patient was diagnosed with orbital pseudotumor. The diagnosis was supported by the presence of orbital inflammation, normal intraocular pressure, absence of optic nerve involvement, and elevated inflammatory markers.

To control the inflammatory response, the patient was initiated on oral corticosteroids (prednisone 60 mg/day). He received counseling regarding the potential side effects of long-term steroid use and was advised to maintain regular follow-up visits to the ophthalmology clinic for monitoring. A tapering schedule for corticosteroid dosage reduction was planned, with close observation for symptom improvement and signs of relapse.

**Figure 2:**
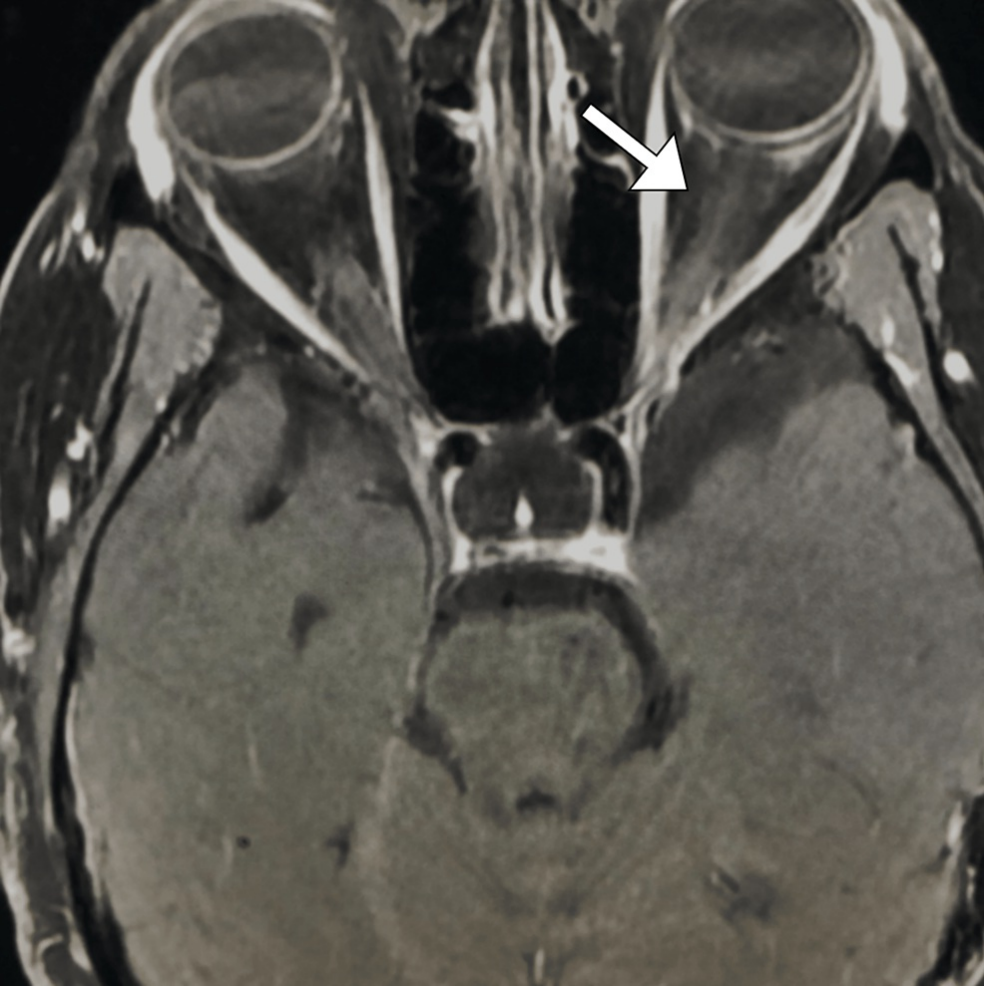
Axial MRI image with contrast demonstrating resolution of previously observed inflammatory changes in the left orbit (arrow). MRI: magnetic resonance imaging

At the one-month follow-up appointment, the patient reported a significant reduction in left eye pain, swelling, and visual disturbances. The prednisone dosage had been gradually reduced to 20 mg/day. Examination revealed decreased eyelid edema, and the visual acuity in the left eye had improved to 20/25. Laboratory investigations, including ESR and CRP, were within normal limits. The patient was advised to continue the tapering schedule and maintain regular follow-up visits to monitor his condition. A repeat MRI study revealed regression of the previously seen inflammatory changes (Figure 2).

## Discussion

Orbital pseudotumor, also known as an idiopathic orbital inflammatory syndrome, is a rare inflammatory condition that poses diagnostic challenges due to its diverse clinical presentation and the absence of a clear etiology [3]. The clinical presentation of orbital pseudotumor can vary widely, making it difficult to differentiate from other orbital pathologies [1]. The most common symptoms reported by patients include eye pain, eyelid swelling, proptosis, diplopia, visual disturbances, and reduced visual acuity. The diagnosis of orbital pseudotumor is based on a combination of clinical findings, imaging studies, and laboratory investigations. Imaging modalities such as MRI with contrast play a crucial role in evaluating the extent and localization of orbital inflammation [3]. It is also highly sensitive in detecting orbital inflammation in patients with orbital pseudotumor, aiding in differentiating it from other orbital pathologies.

Laboratory investigations, including erythrocyte sedimentation rate and C-reactive protein, serve as supportive measures by indicating the presence of systemic inflammation. However, their diagnostic value is limited, as they lack specificity for orbital pseudotumor [4].

The management of orbital pseudotumor centers around controlling the inflammatory response and preserving vision [1,5]. Systemic corticosteroids, such as prednisone, are considered the mainstay of treatment. High-dose oral corticosteroids were found to provide rapid resolution of symptoms and inflammation in patients with orbital pseudotumor [1].

Although the condition can occur at any age, it predominantly affects adults, with a peak incidence in the fourth to sixth decades of life [3]. There is no known gender predilection [3]. While the exact etiology of orbital pseudotumor remains unknown, it is believed to involve an immune-mediated response. Predisposing or risk factors for orbital pseudotumor have not been clearly established [3]. Despite the available literature on orbital pseudotumor, several aspects remain poorly understood. The exact pathogenesis of the condition is still unclear, and there is a need for further research to elucidate the underlying immunological mechanisms involved [4,5]. Additionally, more comprehensive studies are required to assess the long-term outcomes of different treatment modalities and identify predictors of treatment response and relapse [4].

However, the long-term use of corticosteroids is associated with significant side effects, necessitating the exploration of alternative treatment options [1,2]. In recent years, immunosuppressive agents, such as methotrexate and azathioprine, have been utilized as steroid-sparing agents with promising results [2,5]. Biologic therapies targeting specific inflammatory pathways, such as tumor necrosis factor-alpha inhibitors, have also shown efficacy in refractory cases [2].

The role of a biopsy in orbital pseudotumor is a subject of debate [1-3]. The biopsy may be considered in certain situations, such as atypical presentations, suspicion of malignancy, or when other diagnostic modalities fail to provide a definitive diagnosis. However, the decision to perform a biopsy should be individualized and balanced against the potential risks and benefits.

## Conclusions

In conclusion, this case report of orbital pseudotumor presents a comprehensive evaluation of a challenging clinical case, highlighting the diagnostic difficulties and management strategies associated with this rare inflammatory condition. The successful use of oral corticosteroids in controlling the inflammatory response and improving symptoms emphasizes their effectiveness as a primary treatment option. However, the potential risks and side effects associated with long-term steroid use underscore the importance of close follow-up, monitoring, and gradual tapering of medication. By shedding light on the complexities of orbital pseudotumor, this case report provides valuable insights for clinicians faced with similar challenging cases. It contributes to the existing literature by highlighting the significance of a comprehensive approach to diagnosis.
